# Editorial: Obesity and Diabetes: Implications for Brain-Immunometabolism

**DOI:** 10.3389/fnins.2020.00056

**Published:** 2020-02-12

**Authors:** Joana M. Gaspar, Sebastien Talbot, Alexandra Latini

**Affiliations:** ^1^Laboratório de Bioenergética e Estresse Oxidativo - LABOX, Departamento de Bioquímica, Centro de Ciências Biológicas, Universidade Federal de Santa Catarina, Florianópolis, Brazil; ^2^Programa de Pós-Graduação em Bioquímica, Universidade Federal de Santa Catarina, Florianópolis, Brazil; ^3^Département de Pharmacologie et Physiologie, Faculté de Médecine, Université de Montréal, Montréal, QC, Canada

**Keywords:** obesity, energy homeostasis, diabetes, neurodegeneration, inflammation

Obesity affects a quarter of the worldwide population and is a risk factor for numerous pathologies (Gregg and Shaw, 2017). It leads to chronic and systemic low-grade inflammation, which impairs the neuronal circuitries dedicated to controlling the hosts' energy homeostasis. While some of the molecular mechanisms underlying obesity started to unfold, the pathology remains intractable (Valdearcos et al., 2015). The 15 articles composing this collection investigate and discuss crucial mechanistic aspects underlying the neurometabolic changes observed in the central nervous system of obese or diabetic individuals.

Hypothalamic microglia undergo various morphological and functional changes in response to high-fat diet. Initially, the resident microglia are activated and, as diet-induced obesity persists, bone marrow-derived myeloid cells gradually replace the brain resident microglia. Mendes et al. and Macedo et al. reviewed the mechanisms underlying such changes to hypothalamic microglial cells. Also, they assess the various roles played by microglia in this context and how one could target aspects of this cascade to develop therapeutic strategies. Next, Rahman et al. reviewed the mechanisms by which glia–glia crosstalk drive overnutrition-induced hypothalamic inflammation.

Rajchgot and colleagues examined the molecular mechanisms by which chronic hyperglycemia leads to overactive spinal microglia. They also analyzed how such changes sensitized nociceptor neurons and lead to diabetic pain neuropathy (Rajchgot et al.).

Gaspar and Velloso review the major findings that support the involvement of hypoxia-inducible factor in the metabolic and energy regulation during the progression of obesity. Metabolic diseases constitute risk factors for the development of neurodegenerative diseases, although the underlying mechanisms behind such effects are not fully clarified.

de Mello and collaborators reviewed recent evidence linking defects in insulin signaling and autophagy to neurodegeneration and discuss potential interventions targeting these pathways (de Mello et al.). Adipokines mediates the communication between the peripheral and central nervous systems. Forny-Germano et al. reviewed the actions of leptin and adiponectin, with emphasis on how altered signaling of these adipokines lead to cognitive dysfunction and augmented the risk of developing Alzhimer's disease in obese individuals.

Using functional magnetic resonance imaging, Rucker and Ikuta revealed a negative association between body mass index (BMI) and pituitary gland functional connectivity. Their data highlight the importance of dopamine-rich brain regions and the pituitary gland in feeding behavior and body mass gain. Next, Lizarbe et al. analyzed the effect of a high-fat diet on the cortex, the hippocampus, and the hypothalamus metabolic profiles. 1H-magnetic resonance spectroscopy showed that high-fat diet exposed mice have reduced levels of synaptic and glial proteins compromising hippocampal-dependent spatial memory (Lizarbe et al.). Gomes et al. used a rodent model of Parkinson's disease induced by the administration of 6-hydroxydopamine. In addition to the expected motor impairments, they found no impact on the animals' body mass, food intake, and glucose homeostasis.

Inflammation associated with metabolic disorders breakdown the blood–brain-barrier (BBB), increasing its permeability to peripheral immune cells. Van Dyken and Lacoste reviewed the effects of obesity and diabetes-induced inflammation on BBB permeability as well as roles played by leptin and insulin resistance in this process. The authors also discuss the broader implications of neural inflammation, including its connection to Alzheimer's disease, multiple sclerosis, and the gut microbiome. The role of the crosstalk between intestinal immune cells and the enteric nervous system in the control of blood glucose was addressed by Bessac et al. The authors focused on the gut-brain axis as a major pathway for the nutritional stated of the body.

Vagus nerve and brain cholinergic signaling are important in the regulation of metabolic homeostasis and immune function; relevant aspects of this regulation were reviewed with a specific focus on obesity-associated conditions (Chang et al.). In this review, the authors outlined accumulating preclinical evidence for the therapeutic efficacy of cholinergic stimulation in alleviating obesity-associated inflammation, neuroinflammation, and metabolic derangements. Thus, a rationale for further therapeutic developments using pharmacological and bioelectronics cholinergic modulation for clinical benefit was proposed.

Albeit being not long-lasting, pharmacotherapy and bariatric surgeries were demonstrated to be useful tools in the management of morbid obesity. It is well-accepted that lifestyle changes, such as physical exercise, have beneficial effects controlling the BMI, brain energy metabolism, and have neuro-protective effects. The benefits of physical exercise were reviewed by de Oliveira Bristot et al., giving a particular attention on the muscle-neuron crosstalk, mediated by irisin, peroxisome proliferator-activated receptor gamma (PPAR-γ) coactivator 1-alpha (PGC-1α) and mitochondrial uncoupling protein (UCP). Deep brain stimulation (DBS) consists of delivering electrical impulses to specific brain regions modulating neuronal circuit is an approved non-pharmacological therapy for movement disorders. Formolo et al. reviewed the potential effectiveness of DBS for for the treatment of obesity.

## Author Contributions

All authors contributed equally to the writing and editing of the editorial manuscript.

### Conflict of Interest

The authors declare that the research was conducted in the absence of any commercial or financial relationships that could be construed as a potential conflict of interest.
